# Dose escalation based on ^18^F-FDG PET/CT response in definitive chemoradiotherapy of locally advanced esophageal squamous cell carcinoma: a phase III, open-label, randomized, controlled trial (ESO-Shanghai 12)

**DOI:** 10.1186/s13014-022-02099-y

**Published:** 2022-07-29

**Authors:** Hongcheng Zhu, Qiufang Liu, Hao Xu, Miao Mo, Zezhou Wang, Kui Lu, Jialiang Zhou, Junqiang Chen, Xiangpeng Zheng, Jinjun Ye, Xiaolin Ge, Honglei Luo, Qi Liu, Jiaying Deng, Dashan Ai, Shengnan Hao, Junhua Zhang, I Hsuan Tseng, Shaoli Song, Yun Chen, Kuaile Zhao

**Affiliations:** 1 Department of Radiation Oncology, Fudan University Shanghai Cancer Center, Shanghai, China; 2 Department of Oncology, Shanghai Medical College, Fudan University, Shanghai, China; 3 Shanghai Key Laboratory of Radiation Oncology, Shanghai, China; 4 Department of Nuclear Medicine, Fudan University Shanghai Cancer Center, Shanghai, China; 5 Department of Cancer Prevention and Statistics, Fudan University Shanghai Cancer Center, Shanghai, China; 6 Department of Radiation Oncology, Taizhou Second People’s Hospital, Taizhou, Jiangsu China; 7 Department of Radiation Oncology, Affiliated Hospital of Jiangnan University, Wuxi, Jiangsu, China; 8 Department of Radiation Oncology, Fujian Cancer Hospital, Fuzhou, China; 9 Department of Radiation Oncology, Huadong Hospital Affiliated to Fudan University, Shanghai, China; 10 Department of Radiation Oncology, Jiangsu Cancer Hospital, Nanjing, China; 11 Department of Radiation Oncology, The First Affiliated Hospital of Nanjing Medical University, Nanjing, China; 12 Department of Radiation Oncology, The Affiliated Huaian No.1 People’s Hospital of Nanjing Medical University, Huai‘an, Jiangsu China

## Abstract

**Introduction:**

Definitive chemoradiotherapy has established the standard non-surgical treatment for locally advanced esophageal cancer. The standard dose of 50–50.4 Gy has been established decades ago and been confirmed in modern trials. The theorical advantage of better local control and technical advances for less toxicity have encouraged clinicians for dose escalation investigation. ^18^F-fluorodeoxyglucose (^18^F-FDG) positron emission tomography/computed tomography (PET/CT) have the potential to tailor therapy for esophageal patients not showing response to CRT and pioneers the PET-based dose escalation.

**Methods and analysis:**

The ESO-Shanghai 12 trial is a prospective multicenter randomized phase 3 study in which patients are randomized to either 61.2 Gy or 50.4 Gy of radiation dose by PET response. Both groups undergo concurrent chemoradiotherapy with paclitaxel/cisplatin regimen for 2 cycles followed by consolidation chemotherapy for 2 cycles. Patients with histologically confirmed ESCC [T1N1-3M0, T2-4NxM0, TxNxM1 (Supraclavicular lymph node metastasis only), (AJCC Cancer Staging Manual, 8th Edition)] and without any prior treatment of chemotherapy, radiotherapy or surgery against esophageal cancer will be eligible. The primary endpoints included overall survival in PET/CT non-responders (SUV_max_ > 4.0) and overall survival in total population. Patients will be stratified by standardized uptake volume, gross tumor volume and tumor location. The enrollment could be ended, when the number of PET/CT non-responder reached 132 and the total population reached 646 for randomization.

**Ethics and dissemination:**

This trial has been approved by the Fudan University Shanghai Cancer Center Institutional Review Board. Trial results will be disseminated via peer reviewed scientific journals and conference presentations.

*Trial registration* The trial was initiated in 2018 and is currently recruiting patients. *Trial registration number* NCT03790553.

## Background

According to the Global Burden of Disease Study 2019, esophageal cancer (EC) accounted for approximately 535,000 new cancer cases and 498,000 deaths in 2019 worldwide [1]. The 5-year standardized net survival rate ranges between 10 and 30% globally [2]. There are two distinct histopathologic subtypes of EC: esophageal squamous cell carcinoma (ESCC) and esophageal adenocarcinoma. These subtypes vary in terms of incidence, risk factors, location, and age of diagnosis [3], and ESCC is the dominant subtype in Asian populations [4]. Definitive chemoradiotherapy (CRT) has been established as the standard nonsurgical treatment for locally advanced EC. Measures to achieve better local control and overall survival, including radiation dose escalation, chemotherapy regime optimization, and the combination of target agents, have been explored for decades. However, few of these measures have demonstrated positive results in randomized phase III trials. The ESO-Shanghai 12 trial (NCT03790553) was designed to evaluate dose escalation in ESCC chemoradiation based on the response on 18F-fluorodeoxyglucose (18F-FDG) positron emission tomography/computed tomography (PET/CT), with the aim of establishing an approach to precision radiation therapy for EC.

### Standard dose for CRT

The phase III RTOG-8501 trial demonstrated that 50 Gy radiation with concurrent chemotherapy is superior to radiotherapy of 64 Gy alone, thus establishing definitive concurrent CRT as the standard nonsurgical treatment scheme for locally advanced EC [5]. The phase III randomized trial RTOG-9405 compared a high dose of 64.8 Gy and a low dose of 50.4 Gy in definitive concurrent CRT. The results showed that a high dose did not improve survival or local control, with the absolute value of median overall survival and the 2-year survival rate being lower in the high-dose group than in the low-dose group [6]. During the past decade, radiotherapy has entered the era of three-dimensional conformal radiation therapy (3DCRT); however, the standard radiation dose of 50 Gy does not present preferable survival characteristics regardless of whether it is combined with concurrent systemic therapy [7–9]. With the wide application of intensity-modulated radiotherapy (IMRT), radiation oncologists are interested in re-investigating dose escalation in EC. The highly anticipated European CONCORDE [10] and ARTDECO [11] studies, along with a Chinese phase III trial [12], have further demonstrated that increasing the radiation dose for non-selected populations does not offer benefits in definitive CRT for EC in the modern era. However, high doses (≥ 60 Gy) have been widely used in Asia and have demonstrated positive results in Asian populations and ESCC subtypes in retrospective studies [13, 14], and meta-analyses have further contributed to the debate [15, 16]. Moreover, our previous drug comparison phase III trial [17] using involved-field irradiation–based IMRT up to a total dose of 61.2 Gy demonstrated favorable survival outcomes (Table 1).

**Table 1:** Survival outcomes of standard dose or high dose in esophageal cancer dCRT from selected trails

Study (Reference)	Country (Year*)	Pathology	Radiation dose	Radiation technique	Systemic therapies	No. of Patients	2y OS	3y OS	5y OS
RTOG 85–01 [5]	USA (1986–1990)	AC/SCC	50 Gy	2D	DDP + 5-Fu	61	36%	30%	25%
			50 Gy		DDP + 5-Fu	69	36%	30%	25%
			64 Gy		–	62	10%	0%	0%
RTOG 94–05/INT 0123 [6]	USA (1995–1999)	AC/SCC	50.4 Gy	2D	DDP + 5-Fu	109	40%	33%	–
			64.8 Gy		DDP + 5-Fu	109	31%	25%	–
PRODIGE5/ACCORD17 [7]	France (2004–2011)	AC/SCC	50 Gy	3DCRT	FOLFOX	134	–	19.9%	–
					DDP + 5-Fu	133	–	26.9%	–
SCOPE1 [8]	UK (2008–2012)	AC/SCC	50 Gy	3DCRT	CAP + DDP + Cetuximab	129	41.3%	–	–
					CAP + DDP	129	56%	–	–
RTOG 0436 [9]	USA (2008–2013)	AC/SCC	50.4 Gy	3DCRT	PTX + DDP + Cetuximab	159	45%	34%	–
					PTX + DDP	169	44%	28%	–
CONCORDE/PRODIGE26 [10]	France (2011–2019)	AC/SCC	50 Gy	3DCRT/IMRT/VMAT	FOLFOX	109	Median 25.2 m		
			66 Gy			108	Median 23.5 m		
ESO-Shanghai 1 [17]	China (2012–2015)	SCC	61.2 Gy	IMRT	PTX + 5-Fu	217	60.6	55.4%	44.3%
					DDP + 5-Fu	219	61.5%	51.8%	40.8%
ARTDECO [11]	Netherlands (2012–2018)	AC/SCC	50.4 Gy	3DCRT/IMRT	PTX + CBP	130	–	42%	–
			61.6 Gy			130	–	39%	–
Xu et al. [12]	China (2013–2017)	SCC	50 Gy	IMRT	DDP + DTX	153	62.9%	54.0%	–
			60 Gy			152	64.8%	54.1%	–

dCRT = definitive chemoradiotherapy; AC = Adenocarcinoma; SCC = Squamous cell carcinoma; 2D = Two Dimensional; 3DCRT = Threedimensional conformal radiation therapy; IMRT = Intensity-modulated radiation therapy; VMAT = Volumetric-modulated arc therapy; DDP = Cisplatin; 5-Fu = 5-Fluorouracil; FOLFOX = Oxaliplatin + Leucovorin + 5-Fluorouracil; CAP = Capecitabine; PTX = Paclitaxel; CBP = Carboplatin; DTX = Docetaxel; OS = Overall survival

*Patients recruiting year

### Failure patterns of CRT in EC

Researchers have tried to explore failure patterns of EC chemoradiotherapy. Button et al. [18] reported the first failure site in 145 patients receiving chemoradiotherapy for EC. The irradiation field included the gross tumor volume (GTV), plus 3 cm of the proximal and distal normal esophagus and a 1.5-cm margin in the transversal position. Eighty-five patients were identified as treatment failure, including 55 patients with in-field failure, 13 patients with distant metastases, and 14 patients with both. Only 3 patients had relapse observed at the edge of the irradiation field. Welsh et al. [19] retrospectively analyzed 239 patients who received CRT for EC, among whom 50% had local failure and 48% had distant metastases. Of all the cases of local failure, 90% occurred in the GTV, 27% in the clinical target volume (CTV), and 12% in the primary tumor volume (PTV). In the ESO-Shanghai 1 trial, the radiotherapy techniques were involved-field irradiation (no lymph node preventive irradiation) and IMRT, and the radiotherapy dose was 61.2 Gy/34 Fx. Of the 436 trial participants, 258 (59.2%) experienced treatment failure; 37 patients (8.5%) showed failure of irradiation of the lymph nodes in the field, of which 7 cases (1.6%) had simple lymph node failure [20]. Therefore, considering recurrence within the irradiation field accounts for half of cases of CRT failure in EC, dose escalation may be an effective approach to achieve a good prognosis.

### Feasibility and effectiveness of high-dose radiotherapy in EC

The esophagus may be the most important dose-limiting organ. Under the guidance of PET/CT, Yu et al. [21] irradiated more than 50% of the tumor area with the maximum standardized uptake volume (SUV) before radiotherapy. They found that it was safe for patients to receive 70 Gy/25 Fx at the same time and that the short-term toxicity was tolerable. In a phase 1/2 trial, Chen et al. [22] reported that chemoradiotherapy with a simultaneous integrated boost of radiotherapy (at doses of 50.4 Gy to subclinical areas at risk and 63.0 Gy to the gross tumor and involved nodes, all given in 28 fractions) for patients with locally advanced EC was well tolerated, with encouraging local control. If the target volume dose can be increased without affecting the tolerance of normal tissue, local control and the overall therapeutic effect may be improved in EC radiotherapy [23]. In recent years, the progress of radiation technology has ensured the safety and effectiveness of increasing the local radiotherapy dose. Also, because involved-field irradiation entails less toxicity, its use may increase the feasibility of increasing the dose.

### Tumor response assessment

In EC, evaluation of the tumor response to neoadjuvant therapy can not only be used to predict prognosis but also to detect non-responders of CRT and allow adjustment of treatment strategy. According to the MUNICON trial, patients who show a clinical response to induction treatment (chemotherapy- or chemoradiotherapy) have a better prognosis [24]. At present, several methods are available to evaluate the treatment response; among them, ultrasonic gastroscopy and ^18^F-FDG PET/CT have high accuracy [25]. Consequently, improving induction chemotherapy to increase the proportion of treatment responders, as well as intensifying the radiotherapy, could be strategies to improve local disease control or survival. Several phase II trials [26–29] have shown that PET/CT has the potential to tailor therapy for patients not showing an early response to chemotherapy and have pioneered PET-directed EC neoadjuvant therapy (Table 2).

**Table 2:** Selected trails of PET-directed neoadjuvant therapy in esophageal cancer

Study (Reference)	Country (Year*)	Patho logy	PET timing	Metabolic parameters and cutoff	Induction therapy	Arms	Treatment	No. of Patients	pCR	Median OS
MUNICON [24]	Germany (2002–2005)	AC	Day 14	△SUV_max_ ≥ 35%	DDP + CF + 5-FU ± PTX	Responder	Continued nCT + surgery	50	58%	–
						Non-responder	Discontinued nCT + surgery	54	0%	25.8 m
MUNICON II [26]	Germany (2005–2008)	AC	Day 14	△SUV_max_ ≥ 35%	DDP + CF + 5-FU ± PTX	Responder	Continued nCT + surgery	23	36%	–
						Non-responder	Change to nCRT (DDP or 5-Fu + 32 Gy/1.6 Gy bid) + surgery	33	26%	18.3 m
AGITC DOCTOR [27]	Australia (2009–2015)	AC	Day 15	△SUV_max_ ≥ 35%	DDP + 5-Fu	Responder	Continued initial nCT + surgery	45	7%	61 m
						Non-responder	New nCT regime of DCF (DTX + DDP + 5-Fu) + surgery	31	20%	30 m
							Change to nCRT (DCF + 45 Gy/25 Fx) + surgery	34	63%	35 m
CALGB-80803/Alliance [28]	USA (2011–2015)	AC	Day 36—42	△SUV_max_ ≥ 35%	FOLFOX	Responder	nCRT: Continued initial chemo plus RT (50.4 Gy/28 Fx) + surgery	72	37.5%	50.3 m
						Non-responder	nCRT: Crossover to alternative chemo (PTX + CBP) plus RT (50.4 Gy/28 Fx) + surgery	39	19.0%	30.9 m
					PTX + CBP	Responder	nCRT: Continued initial chemo plus RT (50.4 Gy/28 Fx) + surgery	64	12.5%	39.6 m
						Non-responder	nCRT: Crossover to alternative chemo (FOLFOX) plus RT (50.4 Gy/28 Fx) + surgery	50	17.0%	27.6 m
MEMORI [29]	Germany (2014–2018)	AC	Day 14—21	△SUV_max_ ≥ 35%	EOX/XP/mFOLFOX6	Responder	Continued initial nCT + surgery	47	33%	–
						Non-responder	Change to nCRT (PTX + CBP + 41.4 Gy/23 Fx) + surgery	22	55%	–

nCT = neoadjuvant chemotherapy; nCRT = neoadjuvant chemoradiotherapy; PET = Positron Emission Tomography; △SUVmax = The decreased maximum standard uptake values from baseline to PET2; DDP = Cisplatin; CF = Folinc acid; 5-FU = 5-Fluorouracil; PTX = Paclitaxel; FOLFOX = Oxaliplatin + Leucovorin + 5-Fluorouracil; CBP = Carboplatin; EXO = Epirubicin + Capecitabine + Oxaliplatin; XP = Capecitabine + Cisplatin; DCF = Docetaxel + Cisplatin + 5-Fluorouracil; DTX = Docetaxel

*Patients recruiting year

### Rationale for the trial

Based on the above evidence, we designed a phase 3 study to investigate whether an increased dose of 61.2 Gy is superior to the standard dose of 50.4 Gy in definitive chemoradiotherapy of ESCC, especially in patients with no response on ^18^F-FDG PET/CT.

## Methods and analysis

### Design

**Fig. 1:**
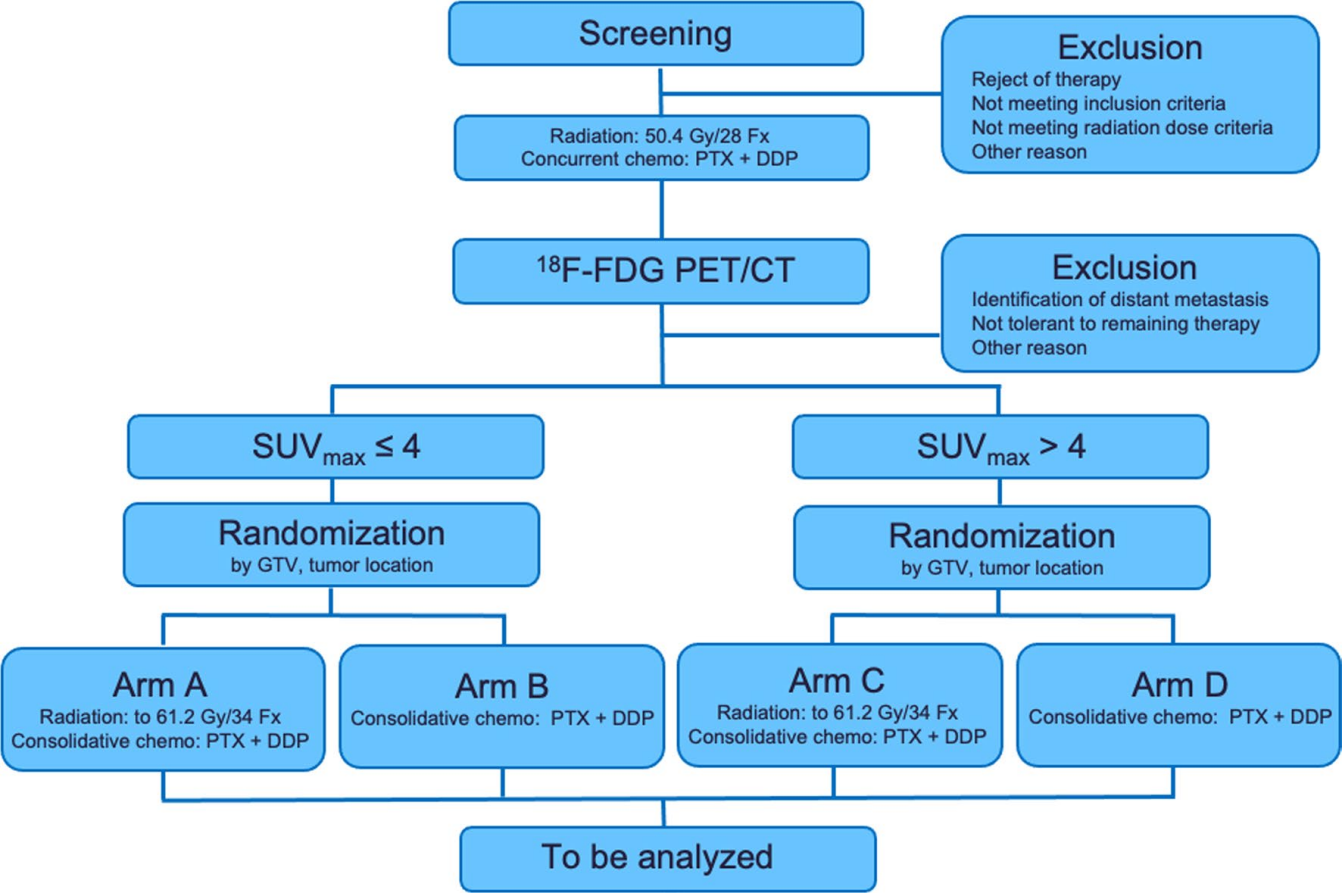
Trial diagram of the ESO-Shanghai 12 trial. PET = Positron emission tomography; SUV = Standard uptake value; GTV = Gross tumor volume; PTX = Paclitaxel; DDP = Cisplatin

The ESO-Shanghai 12 trial is a prospective, multicenter, randomized phase 3 study, in which patients are randomized to receive radiation at a dose of 61.2 Gy or 50.4 Gy based on the ^18^F-FDG PET/CT response (stratified by SUV_max_ > 4 and SUV_max_ ≤ 4). Both arms will undergo concurrent chemoradiotherapy with a paclitaxel/cisplatin (TP) regimen for two cycles followed by consolidation chemotherapy for two cycles (Fig. 1).

### Objectives

#### Primary endpoints

1. Overall survival in PET/CT non-responders (time frame: 2 years)
  The time between the start of the study treatment (day 1) and death from any cause or the last follow-up (for patients who are alive at the end of the study) in patients who have an SUV_max_ > 4 on PET/CT at 28 radiotherapy fractions.
2. Overall survival in the intention-to-treat (ITT) population (time frame: 2 years)
  The time between the start of the study treatment (day 1) and death from any cause or the last follow-up (for patients who are alive at the end of the study) in the ITT population.

#### Secondary endpoints

1. Local control in PET/CT non-responders, the ITT population, and PET/CT responders (time frame: 2 years)
  The time between the start of the study treatment (day 1) and local recurrence (including primary tumor recurrence and regional lymph node failure).
2. Progression-free survival in PET/CT non-responders, the ITT population, and PET/CT responders (time frame: 2 years)
  The time between day 1 and the first event of local failure, metastatic recurrence, progression, or death.
3. Overall survival in PET/CT responders (time frame: 2 years)
  Overall survival in patients who have an SUV_max_ ≤ 4 on PET/CT at 28 radiotherapy fractions.
4. Questionnaire: European Organization for the Research and Treatment of Cancer Quality of Life Questionnaire (EORTC-QLQ)-C30 (time frame: 2 years)
  A quality-of-life score will be obtained based on the answers to the questionnaire.
5. Questionnaire: EORTC-QLQ-OES18 (time frame: 2 years)
  A quality-of-life score will be obtained according to the answers to the questionnaire.
6. Exploration of predictive and prognostic biomarkers.

### Patient selection

#### Inclusion criteria

To be eligible for inclusion in this study, patients must fulfill all the following criteria:

1. Participating in the study voluntarily and able to sign the informed consent form.
2. Aged between 18 and 75 years, and of either sex.
3. Pathologically confirmed with ESCC [T1N1-3M0, T2-4NxM0, TxNxM1 (supraclavicular lymph node metastasis only), (American Joint Committee on Cancer Staging Manual, 8th Edition)].
4. No receipt of radiotherapy, chemotherapy, or other treatments prior to enrollment.
5. Using an effective contraceptive to prevent pregnancy.
6. No severely abnormal hematopoietic, cardiac, pulmonary, renal, or hepatic function, or immunodeficiency.
7. White blood cells (WBC) ≥ 3.5*10^9^/L, hemoglobin ≥ 9 g/dL, neutrophils ≥ 1·5*10^9^/L, platelet count ≥ 100*10^9^/L, alanine aminotransferase (ALAT) and aspartate aminotransferase (ASAT) < 2·5 * upper limit of normal (ULN), total bilirubin (TBIL) < 1·5 * ULN, and creatinine < 1·5 *ULN.
8. An Eastern Cooperative Oncology Group (ECOG) score of 0–2.
9. Life expectancy of more than 3 months.
10. Agrees to undergo ^18^F-FDG PET/CT assessment at 28 radiotherapy fractions.

#### Exclusion criteria

The following patients will be ineligible for this study.

1. Patients whose total radiotherapy dose reaches 61.2 Gy/34 Fx if the normal tissue dose complies with the standard criteria.
2. Patients with esophageal perforation or hematemesis.
3. Patients with a history of radiotherapy or chemotherapy for EC.
4. Patients with a history of surgery within 28 days before Day 1.
5. Patients with a history of prior malignancies (other than skin basal cell carcinoma or cervical carcinoma in situ with disease-free survival of at least 3 years).
6. Patients who are participating in another interventional clinical trial less than 30 days.
7. Pregnant or breastfeeding women or fertile patients who refuse to use contraceptives.
8. Patients with drug addiction, alcoholism, or acquired immunodeficiency (AIDS).
9. Patients experiencing uncontrolled seizures or psychiatric disorders.
10. Patients with any other condition which, in the investigator's opinion, would make them an unsuitable candidate for the clinical trial.

### Study treatment

**Fig. 2:**
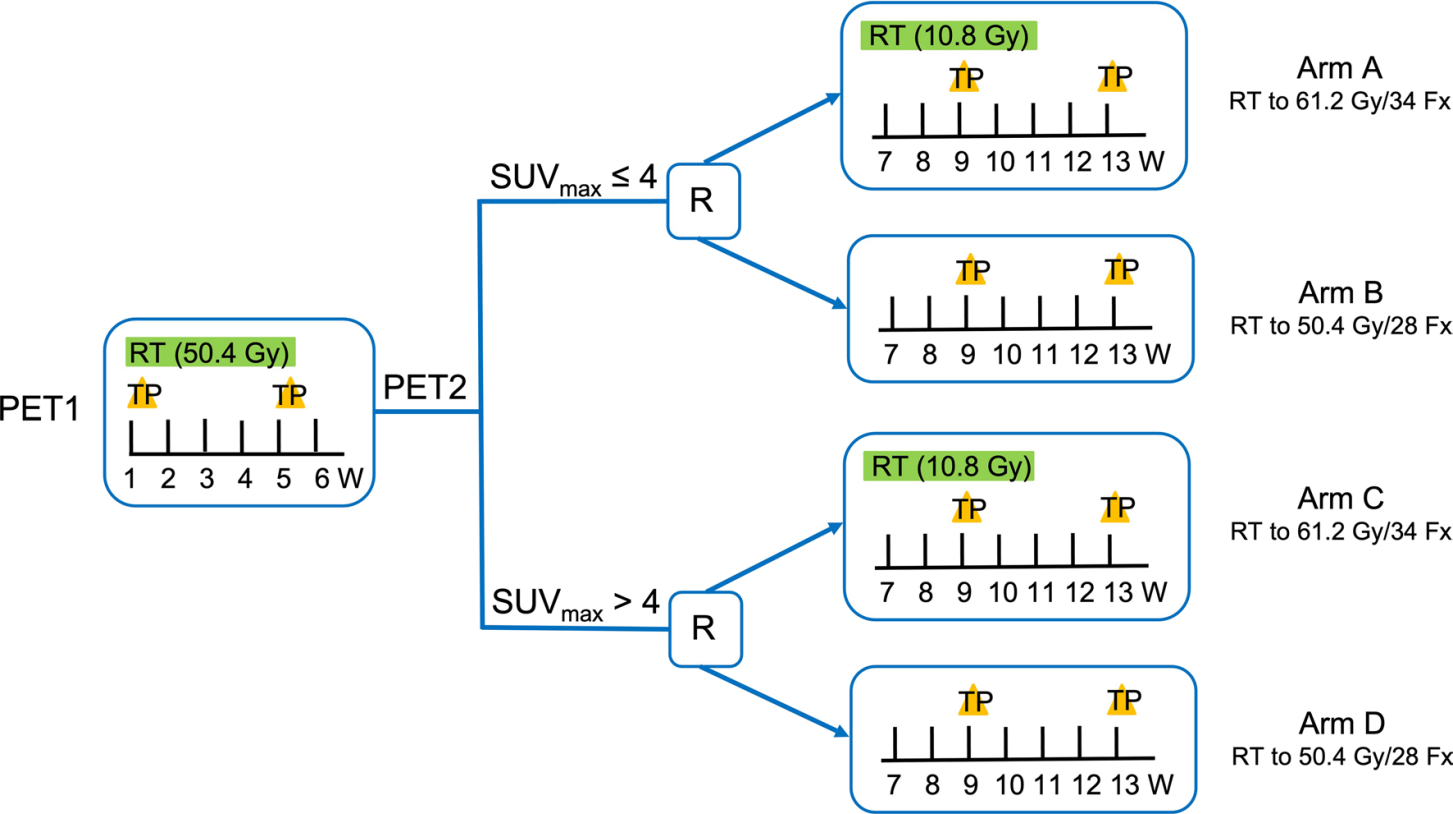
Treatment design of the ESO-Shanghai 12 trial. PET = Positron emission tomography; SUV = Standard uptake value; R = Randomization; RT = Radiation therapy; TP = Paclitaxel + Cisplatin; W = Week

The treatment plan is shown in Fig. 1. Patients will receive radiotherapy combined with concurrent chemotherapy. Radiotherapy will begin on day 1, concurrent with the beginning of cycle 1 of chemotherapy. Radiotherapy will be delivered with photons (≥ 6 MV) to a total dose of 50.4 Gy in 28 fractions or 61.2 Gy in 34 fractions. ^18^F-FDG PET/CT will be performed for all patients at baseline and at 28 radiotherapy fractions (± 3 days), and then patients will be randomized after assessment. Patients will be stratified according to the SUV_max_ (≤ 4 or > 4), GTV (≤ 40 cm^2^ or > 40 cm^2^) and tumor location [cervical/upper thoracic location with heart dose ≤ 10 Gy or (middle/lower thoracic location or heart dose > 10 Gy)] (Fig. 2).

#### ^18^F-FDG PET/CT assessment

1. Pre-PET/CT patient preparation
  Patients will not undergo a barium meal examination at least 1 week before the PET/CT scan.
  Prior to injection, the patient must fast for at least 6 h.
  Blood glucose measurement will be performed before the injection of ^18^F-FDG, and blood glucose levels should be less than 140 mg/dL.
  The height and weight of patients will be measured using calibrated and medically approved devices.
2. Injection of ^18^F-FDG
  Patients will receive an ^18^F-FDG dose of 3.7 MBq/Kg of body weight in accordance with the manufacturer’s recommendations. Patients will be kept in a quiet and dimly lit room before and after the injection.
  A saline flush of 1 mL will follow the ^18^F-FDG injection.
  The exact time of dose calibration will be recorded using a global time recording device, which will be consistently used throughout the study for time recording. The exact time of injection will be noted and recorded to permit the correction of the administered dose for radioactive decay. Furthermore, any of the dose remaining in the tube or syringe, or any ^18^F-FDG that is spilled during the injection will be recorded. The ^18^F-FDG injection will be performed using an intravenous catheter and three-way stopcock.
3. PET/CT imaging
  All PET exams will include three trans-axial, whole-body series, attenuated and non-attenuated, corrected PET and CT images.
  The scan will be conducted 60 ± 10 min after the injection of ^18^F-FDG.
  The patient will be instructed to empty their bladder immediately before the image acquisition.
  A spiral CT scan using the low-dose technique (120 kV, 140 mA, 5-mm slice thickness) will be conducted first, followed by a PET emission scan from the distal femur to the top of the skull.
  A PET emission scan covering the same transverse field of view will be obtained immediately after the CT scan. The PET data will be reconstructed using a Gaussian filter iterative (iterations 4; subsets 8; image size 168) for the reconstruction of emission images. The CT data will be used for attenuation correction of the PET images, and fused images will be displayed on a workstation.
4. Reporting of PET findings and SUV calculations

SUVs are commonly used in clinical practice in addition to visual assessments. The SUV is a measurement of the uptake in a tumor normalized on the basis of a distribution volume. It is calculated as follows:

$${\text{SUV}} = \frac{{{\text{Act}}_{{{\text{voi}}}} \left( {\text{kBq/ml}} \right)}}{{{\text{Act}}_{{{\text{administered}}}} {{\left( {{\text{MBq}}} \right)} \mathord{\left/ {\vphantom {{\left( {{\text{MBq}}} \right)} {{\text{BW}}\left( {{\text{kg}}} \right)}}} \right. \kern-\nulldelimiterspace} {{\text{BW}}\left( {{\text{kg}}} \right)}}}}$$

In this calculation, Act_voi_ is the activity measured in the volume of interest (see “Definitions for volumes of interest (VOI) and regions of interest (ROI)”), Act_administered_ is the administered activity corrected for the physical decay of ^18^F-FDG at the start of acquisition, and BW is body weight. The patient’s height, weight, and sex will be reported to allow for other SUV normalizations (lean body mass, body surface area).

- PET/CT images will be analyzed independently by two senior nuclear medicine physicians using a multimodality computer platform (Syngo, Siemens, Knoxville, TN, USA). For any inconsistent or equivocal interpretations, another experienced radiologist will be invited to the discussion to reach a consensus.

### Radiotherapy

IMRT will be required. The radiation plan for all patients will involve 61.2 Gy/ 34 Fx, at 1.8 Gy/ day for 5 days per week. The standardized-dose group will receive a radiation dose of 50.4 Gy/28 Fx, while the high-dose group will receive 61.2 Gy of radiation. The patients will be immobilized in the supine position and receive a contrast-enhanced planning CT scan 0.5 cm under the cricothyroid membrane (for thoracic tumors) or basis cranii (for cervical tumors) below the kidney. IMRT will be delivered to all patients using 6 MV photons. The radiation target volume will be delineated by the field involved.

### GTV

The GTV will be defined as any visible primary tumor and metastatic lymph nodes. The primary tumor will be delineated using esophagography, esophagoscopy, contrast-enhanced thoracic CT, and ^18^F-FDG PET/CT). Metastatic lymph nodes will be identified using biopsy, increased uptake of FDG on PET/CT, or based on the following radiographic criteria: nodes of ≥ 1 cm in the shortest axis in the intrathoracic and intra-abdominal region or nodes of ≥ 0.5 cm in the shortest axis along the recurrent nerve.

### CTV

The CTV will be defined as the GTV plus 3 cm of the proximal and distal normal esophagus without lateral margins.

### PTV

The PTV will be generated by applying a 1-cm margin to the CTV.

Tissue inhomogeneity correction will be adopted for planning purposes. The criteria for dose distribution will be as follows: 95% of the PTV to receive ≥ 99% of the prescribed dose; 99% of the PTV to receive ≥ 95% of the prescribed dose; < 2 cm^3^ of the PTV to receive ≥ 120% of the prescribed dose; and < 1 cm^3^ of the PTV to receive ≥ 110% of the prescribed dose. The highest and lowest dose points inside the PTV will be recorded.

### Normal organ contouring and dose restrictions

Normal organs, including the spinal cord, heart, and right and left lungs, will be contoured on each slice of the planning CT with no planning margin. The spinal cord dose constraint must not be exceeded for any reason. The heart contours will extend from the beginning of the right atrium and right ventricle (the pulmonary artery trunk, ascending main aorta, and superior vena cava will be excluded) down to the apex of the heart. The lung volume will be defined as the total lung minus the PTV.

The priority order of consideration for normal organ dose restrictions will be as follows:

1. Spinal cord: the highest dose point must be of less than 45 Gy.
2. Lung: the volume of the lung (PTV excluded) receiving 20 Gy must be equal to or less than 30% of the total lung volume, and the mean lung dose must be equal to or less than 15 Gy at the same time.
3. Heart: the mean dose must be less than 40 Gy.

### Dose modifications

It is strongly recommended that the normal organ dose constraints are not exceeded. If any dose constraint needs to be exceeded to achieve adequate coverage of the PTV, the physician will decide whether the dose should be modified, or whether the patient should be excluded from the trial. The acceptable violations of dose modification will be as follows: 92–95% of the PTV to receive ≥ 99% of the prescribed dose and normal organ dose restrictions (except the spinal cord) exceeding 5–10%.

### Radiotherapy interruption

If the any of the following toxicities are observed, radiotherapy will be delayed until the toxicity improves to grade 2 or lower.

- WBC count < 2.0 × 10^9^/L or absolute neutrophil count (ANC) < 1.0 × 10^9^/L.
- Platelets < 50 × 10^9^/L.
- A non-hematological toxicity of grade 3 or higher.

If the following toxicity is observed, radiotherapy will be delayed until complete recovery.

- Mediastinal or thoracic infection with fever over 38.5 °C.

The suspension of radiotherapy will be permitted for 2 weeks at most; if the patient does not recover within 2 weeks, radiotherapy will be terminated.

### Radiotherapy quality assurance

The IMRT planning CT of the first three patients from each participating institution will be sent to Fudan University Shanghai Cancer Center for central quality assurance to ensure that the center complies with the specific study requirements for delineation, planning, and dose distribution.

### Chemotherapy

Patients in both groups will receive two cycles of concurrent chemotherapy with radiotherapy followed by two cycles of consolidation chemotherapy after CRT. Each cycle of chemotherapy will last for 28 days (4 weeks). The drugs to be used include: paclitaxel 135 mg/m^2^/day, intravenously guttae (IVGTT) over 3 h, day 1; and cisplatin 25 mg/m^2^/day, IVGTT, days 1–3.

### Chemotherapy interruption and dose modification

If the following toxicities are observed on day 1, chemotherapy will be delayed until toxicity improves to grade 1 or lower.

- ANC < 1.5 × 10^9^/L.
- Platelets < 70 × 10^9^/L.
- A non-hematological toxicity of grade 2 or higher, except for nausea, vomiting, and alopecia.

A delay to chemotherapy of 2 weeks at most will be allowed; if the patient does not show sufficient improvement within this time, chemotherapy will be terminated.

Chemotherapy dose modifications will be based on the greatest toxicity during the last cycle. Any patients for whom chemotherapy dose modifications are required will receive the modified dose during subsequent cycles. If modifications are needed, the doses of paclitaxel and cisplatin will be decreased by 25% from the planned dose for the first cycle and by 50% for the second cycle. Dose modifications will be permitted twice at most; for patients who still require dose modification after this point, chemotherapy will be terminated.

The criteria for dose modification are as follows:

#### Dose modification of paclitaxel

- Febrile neutropenia (ANC < 0.5 × 10^9^/L and fever over 38.3° C or over 38.0 °C for 1 h).
- Peripheral neuropathy of grade 2 or higher.

#### Dose modification of cisplatin

- Febrile neutropenia (ANC < 0.5 × 10^9^/L and fever over 38.3° C or over 38.0° C for 1 h).
- Peripheral neuropathy of grade 2 or higher.
- Serum creatinine > 3 times the upper limit of normal.

Adverse events will be evaluated according to the National Cancer Institute Common Terminology Criteria for Adverse Events (CTCAE V.4.0). All adverse events that occur during the course of the trial (from the start of treatment until 28 days after the end of treatment), regardless of their relatedness to the study medication, will be recorded. Adverse events that occur more than 28 days after the end of treatment will only be recorded if they are relevant.

### Quality of life

Quality of life (QOL) is measured as secondary endpoints in this trial and will be assessed using hard-copy versions of the EORTC core questionnaire, the EORTC QLQ-C30 (version 3.0) [30], the disease-specific module for EC, and the QLQ-OES18 [31]. The following assessment points have been chosen to describe QOL changes across time: before the start of treatment (baseline), at the end of radiotherapy (28 or 34 fractions), before two consolidative chemotherapy cycles, and at each follow-up. The EORTC-QLQ-C30 and EORTC-QLQ-OES18 questionnaires will be completed by patients and checked by physicians at clinic visits to minimize the number of missing items and assessments. Time windows of ± 3 weeks will be applied for follow-up assessment.

### Adverse events

Adverse events will be accessed according to National Cancer Institute Common terminology for adverse events (NCI-CTCAE) (version 4.0) [32] and will be monitored throughout the study.

### Translational research

The clinical trial will involve the collection of tissue samples and blood samples for future translational research and the development and/or validation of biomarkers. Trial participants will be asked for additional optional consent to participate in this aspect of the study. The standard tissue sample will consist of pretreatment biopsy of tumor tissue. The blood samples will include 2 × 5 ml ethylenediamine tetraacetic acid (EDTA) blood samples, one of which will be collected at the time of pretreatment, postconcurrent and preconsolidation chemotherapy, and the other after recurrence. All samples will be stored at the FUSCC BioBank for future translational research.

### Statistical analysis

For patients with residual tumors of esophagus, it is estimated that the 2-year survival rate of low-dose radiotherapy group and high-dose radiotherapy group is 11% and 27%, according to literature [33, 34]. The expected enrollment time is 7 years, and the last enrolled patients were followed up for 2 years. According to the ratio of 1:1, bilateral α = 0.05, power = 0.80. When 5% of the patients are expected to fall off, 66 cases in each group and 132 cases in total were included in the PET/CT non-responder group. For non-selected population, the 2-year survival rate is estimated to be 40% of the standard-dose radiotherapy group and 49% of the high-dose radiotherapy group, based on literature data [6, 35]. According to the same enrollment and follow-up time conditions, 323 cases are included in each group, and the total number of samples is determined to be 646. The double end-point fixed sequence method was used to test the PET/CT non-responder group first, and then the overall intention-to-treat population. When the number of PET/CT non-responder reached 132 and the total population reached 646, the enrollment could be ended. Fixed sequence is applied in statistics of primary endpoint.

## Discussion

Radiation dose has always been a concern in radiation oncology, in different tumor types and has been debated for several decades [36]. The theorical advantage of better local control and technical advances for less toxicities have encouraged clinicians for dose escalation investigation. Randomized phase III trials have added evidence that high dose does not bring advantage to unselected patients receiving dCRT. At the same time, the evaluation of tumor response by a variety of imaging methods can not only predict the prognosis, but also detect the non-responders and adjust neoadjuvant treatment strategy. In dCRT, the ongoing British SCOPE2 study [37] compare the effects of standard drugs and alternative combinations used in chemotherapy for patients who do not respond to standard drug chemotherapy in the early stage of treatment, as well as compares the effects of standard dose and high-dose radiotherapy. Our current study is dedicated to SCC histology only and compares radiation dose without switching chemotherapy regime. Our response assessment is post radiation rather than early PET response, aiming to investigate the PET-direct radiotherapy in a pure way. Last not least, we abandon elective nodal irradiation and apply involved field irradiation, which is less toxic. The detailed comparison is in Table 3. Esophageal cancer is on the way to the era of immunotherapy, despite the footstone of chemoradiation in locally advanced disease [38–40]. Precise radiotherapy based on biology and imaging will provide new basis for individualized therapies in the future [41].

**Table 3:** Comparison of the ongoing phase III trials of PET-guided dCRT in esophageal cancer: ESO-Shanghai 12 vs SCOPE 2

		ESO-Shanghai 12 (the current study)	SCOPE 2
Country		China	UK
No. of Patients		634	584
Study starting year		2018	2016
Pathology		SCC	AC/SCC
Radiation technique		IMRT	IMRT
Radiation field		IFI	ENI
Chemotherapy prior to PET 2		Concurrent DDP + PTX * 2 cycles	Induction DDP + CAP * 1 cycle
Radiotherapy prior to PET 2		50.4 Gy/28 Fx	No
PET timing		Day of RT to 50.4 Gy/28 Fx ± 3 days	Day 14
Metabolic parameters and cutoff		PET 2 SUV_max_ > 4	△SUV_max_ ≥ 35%
Treatment posterior to intern PET	Responder	RT up to 50.4 Gy/28 Fx + chemotherapy (consolidative DDP + PTX * 2 cycles)	RT (50 Gy/25 Fx) + chemotherapy (concurrent DDP + CAP * 3 cycles)
			RT (60 Gy/25 Fx) + chemotherapy (concurrent DDP + CAP * 3 cycles)
	Non-responder	RT up to 61.2 Gy/34 Fx + chemotherapy (consolidative DDP + PTX * 2 cycles)	RT (50 Gy/25 Fx) + chemotherapy (concurrent weekly PTX + CBP)
			RT (50 Gy/25 Fx) + chemotherapy (concurrent weekly PTX + CBP)
Primary outcomes		2y OS in PET/CT non-responder	2y TIFFS in SCC
		2y OS in all subjects	2y OS in SCC
		–	2y TIFFS in SCC switching chemotherapy
		–	2y TIFFS in AC
		–	2y TIFFS in AC switching chemotherapy

PET = Positron emission tomography; PET/CT = Positron emission tomography/Computational tomography; dCRT = definitive chemoradiotherapy; AC = Adenocarcinoma; SCC = Squamous cell carcinoma; IMRT = Intensity-modulated radiation therapy; IFI = Involved-field irradiation; ENI = Elective nodal irradiation; DDP = Cisplatin; PTX = Paclitaxel; CAP = Capecitabine; CBP = Carboplatin; RT = Radiation therapy; TIFFS = Treatment failure free survival; OS = Overall survival

## Data Availability

Data sharing not applicable to this article as no datasets were generated or analyzed during the current study until now.

## Abbreviations

dCRT: definitive chemoradiotherapy
AC: Adenocarcinoma
SCC: Squamous cell carcinoma
2D: Two dimensional
3DCRT: Three-dimensional conformal radiation therapy
IMRT: Intensity-modulated radiation therapy
VMAT: Volumetric-modulated arc therapy
DDP: Cisplatin
5-Fu: 5-Fluorouracil
FOLFOX: Oxaliplatin + Leucovorin + 5-Fluorouracil
CAP: Capecitabine
PTX: Paclitaxel
CBP: Carboplatin
DTX: Docetaxel
OS: Overall survival
SUV: Standard uptake value
GTV: Gross tumor volume
nCT: Neoadjuvant chemotherapy
nCRT: Neoadjuvant chemoradiotherapy
PET: Positron emission tomography
△SUV_max_: The decreased maximum standard uptake values from baseline to PET2
CF: Folinc acid
EXO: Epirubicin + Capecitabine + Oxaliplatin
XP: Capecitabine + Cisplatin
DCF: Docetaxel + Cisplatin + 5-Fluorouracil
R: Randomization
RT: Radiation therapy
W: Week
PET/CT: Positron emission tomography/Computational tomography
IFI: Involved-field irradiation
ENI: Elective nodal irradiation
TIFFS: Treatment failure free survival

## References

[1] Kocarnik JM, Compton K, Global Burden of Disease 2019 Cancer Collaboration (2021). Cancer incidence, mortality, years of life lost, years lived with disability, and disability-adjusted life years for 29 cancer Groups From 2010 to 2019: a systematic analysis for the global burden of disease study 2019. JAMA Oncol.

[2] Allemani C, Matsuda T, Di Carlo V (2018). Global surveillance of trends in cancer survival 2000–14 (CONCORD-3): analysis of individual records for 37 513 025 patients diagnosed with one of 18 cancers from 322 population-based registries in 71 countries. Lancet.

[3] GBD 2017 Oesophageal Cancer Collaborators (2020). The global, regional, and national burden of oesophageal cancer and its attributable risk factors in 195 countries and territories, 1990–2017: a systematic analysis for the global burden of disease study 2017. Lancet Gastroenterol Hepatol.

[4] Abnet CC, Arnold M, Wei WQ (2018). Epidemiology of esophageal squamous cell carcinoma. Gastroenterology.

[5] Cooper JS, Guo MD, Herskovic A (1999). Chemoradiotherapy of locally advanced esophageal cancer: long-term follow-up of a prospective randomized trial (RTOG 85–01). Radiat Ther Oncol Gr JAMA.

[6] Minsky BD, Pajak TF, Ginsberg RJ (2002). INT 0123 (radiation therapy oncology group 94–05) phase III trial of combined-modality therapy for esophageal cancer: high-dose versus standard-dose radiation therapy. J Clin Oncol Off J Am Soc Clin Oncol.

[7] Conroy T, Galais MP, Raoul JL (2014). Definitive chemoradiotherapy with FOLFOX versus fluorouracil and cisplatin in patients with oesophageal cancer (PRODIGE5/ACCORD17): final results of a randomised, phase 2/3 trial. Lancet Oncol.

[8] Crosby T, Hurt CN, Falk S (2013). Chemoradiotherapy with or without cetuximab in patients with oesophageal cancer (SCOPE1): a multicentre, phase 2/3 randomised trial. Lancet Oncol.

[9] Suntharalingam M, Winter K, Ilson D (2017). Effect of the addition of cetuximab to paclitaxel, cisplatin, and radiation therapy for patients with esophageal cancer: the NRG oncology RTOG 0436 phase 3 randomized clinical trial. JAMA Oncol.

[10] Crehange G, Mvondo C, Bertaut A, Pereira R, Rio E, Peiffert D, Gnep K, Benezery K, Ronchin P, Noel G, Mineur L, Drouillard A, Blanc J, Rouffiac M, Boustani J (2021). Exclusive chemoradiotherapy with or without radiation dose escalation in esophageal cancer: multicenter phase 2/3 randomized trial CONCORDE (PRODIGE-26). Int J Radiat Oncol Biol Phys.

[11] Hulshof MCCM, Geijsen ED, Rozema T (2021). Randomized study on dose escalation in definitive chemoradiation for patients with locally advanced esophageal cancer (ARTDECO Study). J Clin Oncol.

[12] Xu YJ, Zhu WG, Liao ZX (2020). A multicenter randomized prospective study of concurrent chemoradiation with 60 Gy versus 50 Gy for inoperable esophageal squamous cell carcinoma. Zhonghua Yi Xue Za Zhi.

[13] Brower JV, Chen S, Bassetti MF (2016). Radiation dose escalation in esophageal cancer revisited: a contemporary analysis of the national cancer data base, 2004 to 2012. Int J Radiat Oncol Biol Phys.

[14] Chang CL, Tsai HC, Lin WC (2017). Dose escalation intensity-modulated radiotherapy-based concurrent chemoradiotherapy is effective for advanced-stage thoracic esophageal squamous cell carcinoma. Radiother Oncol.

[15] Luo HS, Huang HC, Lin LX (2019). Effect of modern high-dose versus standard-dose radiation in definitive concurrent chemo-radiotherapy on outcome of esophageal squamous cell cancer: a meta-analysis. Radiat Oncol.

[16] Sun X, Wang L, Wang Y, Kang J, Wei Jiang Y, Men ZH (2020). High vs. low radiation dose of concurrent chemoradiotherapy for esophageal carcinoma with modern radiotherapy techniques: a meta-analysis. Front Oncol.

[17] Chen Y, Ye J, Zhu Z (2019). Comparing paclitaxel plus fluorouracil versus cisplatin plus fluorouracil in chemoradiotherapy for locally advanced esophageal squamous cell cancer: a randomized, multicenter, phase III clinical trial. J Clin Oncol.

[18] Button MR, Morgan CA, Croydon ES (2009). Study to determine adequate margins in radiotherapy planning for esophageal carcinoma by detailing patterns of recurrence after definitive chemoradiotherapy. Int J Radiat Oncol Biol Phys.

[19] Welsh J, Settle SH, Amini A (2012). Failure patterns in patients with esophageal cancer treated with definitive chemoradiation. Cancer.

[20] Zhu H, Rivin Del Campo E (2021). Involved-field irradiation in definitive chemoradiotherapy for locoregional esophageal squamous cell carcinoma: results from the ESO-Shanghai 1 Trial. Int J Radiat Oncol Biol Phys.

[21] Yu W, Cai XW, Liu Q (2015). Safety of dose escalation by simultaneous integrated boosting radiation dose within the primary tumor guided by (18)FDG-PET/CT for esophageal cancer. Radiotherapy and oncology : journal of the European Society for Therapeutic Radiology and Oncology.

[22] Chen D, Menon H, Verma V (2019). Results of a phase 1/2 trial of chemoradiotherapy with simultaneous integrated boost of radiotherapy dose in unresectable locally advanced esophageal cancer. JAMA Oncol.

[23] Warren S, Partridge M, Carrington R (2014). Radiobiological determination of dose escalation and normal tissue toxicity in definitive chemoradiation therapy for esophageal cancer. Int J Radiat Oncol Biol Phys.

[24] Lordick F, Ott K, Krause BJ (2007). PET to assess early metabolic response and to guide treatment of adenocarcinoma of the oesophagogastric junction: the MUNICON phase II trial. Lancet Oncol.

[25] Noordman BJ, Spaander MCW, Valkema R (2018). Detection of residual disease after neoadjuvant chemoradiotherapy for oesophageal cancer (preSANO): a prospective multicentre, diagnostic cohort study. Lancet Oncol.

[26] zum Büschenfelde CM, Herrmann K, Schuster T (2011). (18)F-FDG PET-guided salvage neoadjuvant radiochemotherapy of adenocarcinoma of the esophagogastric junction: the MUNICON II trial. J Nucl Med.

[27] Barbour AP, Walpole ET, Mai GT (2020). Preoperative cisplatin, fluorouracil, and docetaxel with or without radiotherapy after poor early response to cisplatin and fluorouracil for resectable oesophageal adenocarcinoma (AGITG DOCTOR): results from a multicentre, randomised controlled phase II trial. Ann Oncol.

[28] Goodman KA, Ou FS, Hall NC, Bekaii-Saab T, Fruth B, Twohy E, Meyers MO, Boffa DJ, Mitchell K, Frankel WL, Niedzwiecki D, Noonan A, Janjigian YY, Thurmes PJ, Venook AP, Meyerhardt JA, O'Reilly EM, Ilson DH (2021). Randomized phase II study of PET response-adapted combined modality therapy for esophageal cancer: mature results of the CALGB 80803 (Alliance) trial. J Clin Oncol.

[29] Lorenzen S, Quante M (2019). PET-directed combined modality therapy for gastroesophageal junction cancer: first results of the prospective MEMORI trial. J Clin Oncol.

[30] Aaronson N, Ahmedzai S, Bergman B (1993). The European organization for research and treatment of cancer QLQ-C30: a quality-of-life instrument for use in international clinical trials in oncology. J Natl Cancer Inst.

[31] Blazeby JM, Conroy T, Hammerlid E (2003). Clinical and psychometric validation of an EORTC questionnaire module, the EORTC QLQ-OES18, to assess quality of life in patients with oesophageal cancer. Eur J Cancer.

[32] US Department of Health and Human Services. Common toxicology criteria (Common Terminology Criteria for Adverse Events [CTCAE] V4.03. 2010. https://evs.nci.nih.gov/ftp1/CTCAE/CTCAE_4.03/CTCAE_4.03_2010-06-14_QuickReference_5x7.pdf. Accessed 19 May 2013.

[33] Monjazeb AM, Riedlinger G, Aklilu M, Geisinger KR, Mishra G, Isom S, Clark P, Levine EA, William Blackstock A (2010). Outcomes of patients with esophageal cancer staged with [^18^F] Fluorodeoxyglucose positron emission tomography (FDG-PET): Can postchemoradiotherapy FDG-PET predict the utility of resection?. J Clin Oncol.

[34] Cuenca X, Hennequin C, Hindié E (2013). Evaluation of early response to concomitant chemoradiotherapy by interim 18F-FDG PET/CT imaging in patients with locally advanced oesophageal carcinomas. Eur J Nucl Med Mol Imaging.

[35] Chen Y, Zhang Z, Jiang G (2016). Gross tumor volume is the prognostic factor for squamous cell esophageal cancer patients treated with definitive radiotherapy. J Thorac Dis.

[36] Lee NY, Zhang Q, Pfister DG (2012). Addition of bevacizumab to standard chemoradiation for locoregionally advanced nasopharyngeal carcinoma (RTOG 0615): a phase 2 multi-institutional trial. Lancet Oncol.

[37] Gwynne S, Higgins E, Poon King A (2019). Driving developments in UK oesophageal radiotherapy through the SCOPE trials. Radiat Oncol.

[38] Kelly RJ, Ajani JA, Kuzdzal J (2021). Adjuvant nivolumab in resected esophageal or gastroesophageal junction cancer. N Engl J Med.

[39] Shah MA, Bennouna J, Doi T, Shen L, Kato K, Adenis A, Mamon HJ, Moehler M, Xiaolong F, Cho BC, Bordia S, Bhagia P, Shih C-S, Desai A, Enzinger P (2021). KEYNOTE-975 study design: a phase III study of definitive chemoradiotherapy plus pembrolizumab in patients with esophageal carcinoma. Futur Oncol.

[40] Yu R, Wang W, Li T (2021). RATIONALE 311: tislelizumab plus concurrent chemoradiotherapy for localized esophageal squamous cell carcinoma. Futur Oncol.

[41] Scott JG, Sedor G, Ellsworth P (2021). Pan-cancer prediction of radiotherapy benefit using genomic-adjusted radiation dose (GARD): a cohort-based pooled analysis. Lancet Oncol.

